# Effects of a Mealworm (*Tenebrio molitor*) Extract on Metabolic Syndrome-Related Pathologies: In Vitro Insulin Sensitivity, Inflammatory Response, Hypolipidemic Activity and Oxidative Stress

**DOI:** 10.3390/insects13100896

**Published:** 2022-09-30

**Authors:** Joaquín Navarro del Hierro, Emma Cantero-Bahillo, M. Teresa Fernández-Felipe, Mónica R. García-Risco, Tiziana Fornari, Patricia Rada, Laura Doblado, Vitor Ferreira, Ana B. Hitos, Ángela M. Valverde, María Monsalve, Diana Martin

**Affiliations:** 1 Departamento de Producción y Caracterización de Nuevos Alimentos, Instituto de Investigación en Ciencias de la Alimentación (CIAL) (CSIC–UAM), 28049 Madrid, Spain; joaquin.navarrodel@uam.es (J.N.d.H.); emma.cantero@uam.es (E.C.-B.); mariateresa.fernandezf@uam.es (M.T.F.-F.); monica.rodriguez@uam.es (M.R.G.-R.); tiziana.fornari@uam.es (T.F.); 2 Sección Departamental de Ciencias de la Alimentación, Facultad de Ciencias, Universidad Autónoma de Madrid, 28029 Madrid, Spain; 3 Instituto de Investigaciones Biomédicas Alberto Sols (CSIC-UAM), 28049 Madrid, Spain; prada@iib.uam.es (P.R.); lauradoblado@iib.uam.es (L.D.); vdasilva@iib.uam.es (V.F.); ahitos@iib.uam.es (A.B.H.); avalverde@iib.uam.es (Á.M.V.); mpmonsalve@iib.uam.es (M.M.); 4 Centro de Investigación Biomédica en Red de Diabetes y Enfermedades Metabólicas Asociadas (CIBERDEM), Instituto de Salud Carlos III, 28029 Madrid, Spain

**Keywords:** *Tenebrio molitor*, bioactive extract, DPPH, pancreatic lipase, cholesterol, mitochondrial respiration, inflammation, insulin sensitivity, reactive oxygen species

## Abstract

**Simple Summary:**

Among edible insects, mealworm has gained attention due to its proven beneficial effects; however, mealworm products other than flour, such as concentrated forms or extracts different to proteins, are still rare. This novel food source can have a positive impact on multiple diseases such as via the metabolic syndrome. Our objective was to describe the composition of a mealworm extract and study its effect on metabolic syndrome-related pathologies. The extract interfered with the absorption of cholesterol and partially blocked the digestion of fats. It also reduced the inflammatory response, enhanced the sensitivity to insulin and showed strong antioxidant potential. Mealworm extract might be an interesting candidate for ameliorating the risk factors associated with the metabolic syndrome.

**Abstract:**

The mealworm *(Tenebrio molitor* Linnaeus 1758) is gaining importance as one of the most popular edible insects. Studies focusing on its bioactivities are increasing, although alternative forms of consumption other than the whole insect or flour, such as bioactive non-protein extracts, remain underexplored. Furthermore, the incidence of metabolic syndrome-related pathologies keeps increasing, hence the importance of seeking novel natural sources for reducing the impact of certain risk factors. The aim was to study the potential of a non-protein mealworm extract on metabolic syndrome-related pathologies, obtained with ethanol:water (1:1, *v*/*v*) by ultrasound-assisted extraction. We characterized the extract by gas-chromatography mass-spectrometry and assessed its hypolipidemic potential, its ability to scavenger free radicals, to attenuate the inflammatory response in microglial cells, to affect mitochondrial respiration and to enhance insulin sensitivity in mouse hepatocytes. The extract contained fatty acids, monoglycerides, amino acids, certain acids and sugars. The mealworm extract caused a 30% pancreatic lipase inhibition, 80% DPPH· scavenging activity and 55.9% reduction in the bioaccessibility of cholesterol (*p =* 0.009). The extract was effective in decreasing iNOS levels, increasing basal, maximal and ATP coupled respiration as well as enhancing insulin-mediated AKT phosphorylation at low insulin concentrations (*p* < 0.05). The potential of a non-protein bioactive mealworm extract against metabolic syndrome-related pathologies is shown, although further studies are needed to elucidate the mechanisms and relationship with compounds.

## 1. Introduction

Metabolic syndrome (MetS) is an early sign of the future progression of chronic diseases such as type 2 diabetes and/or obesity. It is characterized by the combination of three or more cardiovascular risk factors (hypertension, hyperglycemia, dyslipidemia and abdominal obesity) [1]. Along with lifestyle and dietary modifications, MetS-related risk factors are commonly treated through pharmacological approaches; however, these treatments often have unpleasant side effects, and hence, novel natural compounds or extracts with less side effects are being continuously searched for. 

Plants, fungi and algae, among others, have been widely demonstrated to be very well-known natural sources rich in diverse bioactive compounds of different chemical natures, and have been further demonstrated to exhibit diverse bioactivities against MetS such as anti-inflammatory, antihypertensive, antioxidant, anticarcinogenic, antidiabetic and anti-obesity activities [2,3,4]. However, many other natural sources, such as edible insects, have not been fully explored but have started to receive overwhelming attention in recent years. In this regard, edible insects gained enormous popularity when the Food and Agriculture Organization (FAO) and other world authorities recommended the inclusion of insects in our diets in 2013 [5]. The progressive increase in world population in the coming years and the parallel problem of a potential shortage of proteins of animal origin to supply world demand have motivated such recommendations to search for alternative food sources. In this sense, edible insects are currently one of the most adequate candidates, given that the nutritional requirements for lipids, proteins, or other micronutrients are fully covered by this novel source. These have been shown to be a sustainable food with a low environmental impact, an important additional advantage of current necessity [6,7]. Subsequently, the recommendations given by the FAO had a key impulse when the European Food Safety Authority (EFSA) considered whole insects and their parts as novel foods from 1st January 2018 for the first time, based on the new Regulation (EU) 2015/2283 [8]. One of the most important milestones in the edible insects field occurred in January 2021 with the favorable ruling of the first application of a novel food made from insects, specifically from *Tenebrio molitor* larvae, commonly referred to as mealworms. A positive opinion was issued by the EFSA concerning the safe intake of this edible insect under the proposed uses and levels [9] after being authorized by the European Commission in June 2021. Since then, two more insect species were approved, with more species likely to come in the following months.

A great and progressive increase in studies centered around the potential biological properties of edible insects has occurred in recent years, aiming to elucidate the entire potential of these novel foods previously evidenced through what is known as ‘entomotherapy’. Many such studies suggest that edible insects have the potential to be transformed into functional food, bioactive ingredients or nutraceuticals [10]. As such, multiple bioactivities are being widely and deeply elucidated for edible insects, including their antilipidemic, anti-inflammatory, antiproliferative, antioxidant, antimicrobial, antihypertensive or antiangiogenic potential [11,12,13,14,15]. In the specific case of *T. molitor,* multiple studies have already demonstrated certain effects on metabolic syndrome. However, many of these bioactivities have been linked to either the whole insect in the form of flour, or other fractions such as proteins, lipids and chitin [10]. Therefore, it remains of interest to explore how other presentations of mealworm, such as extracts, can modulate different metabolic syndrome-related pathologies. In this sense, we previously demonstrated the antioxidant and pancreatic-lipase inhibitory activities of non-protein extracts obtained under advanced extraction technologies from different species of edible insects, including *T. molitor* [16,17,18]. Recently, Ham et al. [19] tested the effect of a mealworm fermentation extract in diet-induced obese mice and found improvements in reducing obesity, steatosis and whole-body glucose homeostasis. However, no mechanistic insights related to the beneficial effects were assessed.

The aim of this work was to study the potential of a non-protein extract obtained from *T. molitor* larvae for different pathologies related to metabolic syndrome, with a special focus on diabetes and dyslipidemia. The effect of this extract on mitochondrial respiration, mitochondrial superoxide production, inflammation and insulin sensitivity was evaluated by different assays in cell systems, and the inhibition of pancreatic lipase and cholesterol absorption was evaluated by models of in vitro gastrointestinal digestion. Additionally, the mealworm extract was characterized by gas chromatography in order to elucidate its composition.

## 2. Materials and Methods

### 2.1. Raw Materials and Chemicals

Dried larvae from *T. molitor* Linnaeus 1758 were purchased from a local supplier. Absolute ethanol (131086.1214) and sodium carbonate (131648.1210) were purchased from Panreac (Barcelona, Spain). Methanol (6712–25) and dimethyl sulfoxide (DMSO) (LC1334) were purchased from Macron Fine Chemicals (Gliwice, Poland) and Lab-Scan (Dublin, Ireland), respectively. N,O-bis-(trimethylsilyl)trifluoroacetamide (BSTFA) (15238), 2,2-diphenyl-1-picrylhydrazyl (DPPH˙) (257621), Dulbecco’s phosphate buffered saline (PBS) (59300C), lipase from porcine pancreas (L3126) and 4-methylumbelliferyl oleate (4-MUO) (75164) were purchased from Sigma-Aldrich Chemie GmbH (Steinheim, Germany).

### 2.2. Production of the Mealworm Extract by Ultrasound-Assisted Extraction

Prior to extraction, larvae were ground in a knife mill (Grindomix GM 200, Retsch GmbH, Haan, Germany), kept in sealed bags and stored at room temperature protected from oxygen, light and moisture until submitted to extraction. Extractions were carried out for 15 min by direct sonication (Branson SFX250 Digital Sonifier, Branson Ultrasonics, Danbury, CT, USA) with an ultrasonic probe (1/2″ diameter) at a sonication output amplitude of 60% at 20 kHz, as described by Navarro del Hierro et al. [16]. A mixture of ethanol and milliQ water (1:1, *v*/*v*) was used as an extraction solvent at a sample/solvent ratio of 1:10 (*w*/*v*). The temperature during the extraction process was kept under 70 °C. Then, the mixture was centrifuged at 2688× *g* rpm for 10 min. The whole extraction procedure was performed in quintuplicate and all the supernatants were then combined into a single one. Ethanol contained in the supernatant was dried under vacuum using a rotary evaporator (Heidolph Instruments, Schwabach, Germany). Once ethanol was removed from the supernatant, the remaining aqueous fraction was freeze-dried. The extraction yield was then calculated as the weight of extract with respect to the total initial weight of the ground insect sample (considering the five extractions) expressed in percentage.

### 2.3. Analysis of the Mealworm Extract by Gas Chromatography-Mass Spectrometry (GC-MS)

The mealworm extract was characterized in an Agilent 7890A GC-MS (Agilent Technologies, Santa Clara, CA, USA) as described by Navarro del Hierro et al. [16], and the previous derivatization of the samples by silylation with BSTFA at 10 mg/mL (60 min, 75 °C). The equipment comprised a split/splitless injector, G4513A autoinjector, an electronic pressure control and a 5975C triple-axis mass spectrometer detector. The column employed was an Agilent HP-5MS capillary column (30 m × 0.25 mm i.d., 0.25 µm-phase thickness) and the carrier gas was helium at a flow of 2 mL/min. Sample injections (1 µL) were performed in splitless mode. The injector temperature was 260 °C and the mass spectrometer ion source and interface temperatures were 230 and 280 °C, respectively. The temperature of the oven was initiated at 50 °C, held for 3 min and increased at a rate of 15 °C/min to 310 °C, which was then held for 25 min. The mass spectra were obtained by electronic impact at 70 eV. The scanning speed was 1.6 scans/s in a mass range of 30–700 amu. Identification of compounds was performed by the NIST MS data library, by the mass spectra according to literature, or according to commercial standards (most fatty acids, glycerides, sugars and sterols) previously derivatized following the same procedure as samples.

### 2.4. Pancreatic Lipase Inhibition Assay

The inhibitory activity of the mealworm extract against the pancreatic lipase was assayed by using 4-MUO as substrate, according to Herrera at al. [20]. The assay was performed under simulated intestinal conditions by using a buffer (Trizma-Maleic 100 mM pH 7.5, 0.15 M NaCl, and 5.1 mM CaCl_2_) with bile salts (7.81 mg/mL) and lecithin (3.12 mg/mL). The reaction mixture consisted of 0.5 mL of extract in digestion buffer solution at different concentrations, 0.5 mL of freshly prepared pancreatic lipase at 1 mg/mL (0.01 g of lipase in 10 mL digestion buffer, stirred for 10 min and centrifuged for 10 min at 2688 × *g*), and 1 mL of 4-MUO solution at 0.1 mM in digestion buffer. The final concentration of the extract in the mixture ranged between 0.0625 and 1.5 mg/mL. The control samples were prepared following the same procedure in absence of extract, and extract controls were prepared in the absence of lipase and 4-MUO. The reaction mixture was placed in an orbital incubator (Titramax 1000, Heidolph Instruments, Schwabach, Germany) at 37 °C with shaking (250 rpm) for 20 min and protected from the light. After incubation, three aliquots of 150 µL were added to a 96-well plate. The amount of 4-MUO hydrolyzed by lipase in the presence of the extracts was measured using an Infinite M200 fluorescence microplate reader (Tecan, Salzburg, Austria) at an excitation wavelength of 350 ± 10 nm and an emission wavelength of 450 nm. The inhibition of the pancreatic lipase by the extracts was calculated using the following formula:

% Inhibition = 100 − [(F_extract sample_ − F_extract control_)/F_control sample_] × 100

where F_extract sample_ is the fluorescence of the reaction with extract, F_extract control_ is the fluorescence of the extract controls in absence of enzyme and substrate, and F_control sample_ is the fluorescence of the reaction mixture without extract. Determination was performed in triplicate.

### 2.5. Effect of the Mealworm Extract on the Intestinal Bioaccessibility of Cholesterol

The evaluation of the potential hypocholesterolemic effect of the mealworm extract was performed according to Navarro del Hierro et al. [21] by simulating the in vitro gastrointestinal digestion of cholesterol in the presence and absence of the extracts.

A lipid mixture containing 3 mg of lecithin, 8 mg of cholesterol and 80 mg of refined olive oil was prepared in order to simulate a typical mixture of dietary lipids under the form of triglycerides, phospholipids and cholesterol, at proportions that can be found in dietary fats [21]. Then, 5 or 10 mg of extracts was added. Subsequently, 2.2 mL of gastric solution at pH 2.5 (150 mM NaCl, 6 mM CaCl_2_ and 0.1 mM HCl) was added. A negative control (in the absence of extracts) and a positive control (containing β-sitosterol at the same concentrations as extracts) were also prepared. Then, the mixture was gently stirred at 250 rpm in an orbital shaker at 37 °C for 1 min to allow the dispersion of the components. The gastric digestion started after the addition of 0.225 mL of a fresh extract of gastric enzymes containing gastric lipase (16 mg/mL) and pepsin (29.4 mg/mL) in gastric solution previously stirred for 10 min. The reaction was performed for 45 min and 250 rpm. Then, for the intestinal digestion, 0.95 mL of a solution simulating biliary secretion were added (0.05 g of lecithin, 0.125 g of bile salts, 0.25 mL of 325 mM CaCl_2_ solution, 0.75 mL of 3.25 M NaCl solution and 5 mL of Trizma-maleate buffer 100 mM pH 7.5, stirred for 10 min) and the whole medium was stirred for 1 min at 37 °C. The intestinal digestion was initiated by the addition of 0.225 mL of a fresh pancreatin extract at 15.6 mg/mL in trizma-maleate buffer, which was previously stirred for 10 min and centrifuged at 2688× *g* for 15 min. The reaction was performed for 60 min.

Once the digestion concluded, the whole medium was submitted to centrifugation at 2688× *g* for 40 min. After centrifugation, the aqueous micellar phase, which contained the solubilized cholesterol, was collected. The digestion of each sample was performed at least in duplicate.

For the determination of the bioaccessibility of cholesterol, two sets of digestions were prepared in duplicate. One, which was the whole digestion medium and the second one, the isolated micellar phase. Then, each of the digestions were vortex-extracted for 1 min with ethyl acetate at a ratio of 1:1 and centrifuged for 10 min at 2688× *g*. The top phase was collected and the bottom phase was extracted again with the same volume of ethyl acetate under the described conditions. Cholesterol in the collected phases was analyzed by HPLC-DAD, according to Kolarič and Šimko with slight modifications [22]. Briefly, a LC-2030C 3D Plus system (Shimadzu, Kyoto, Japan) equipped with a quaternary pump and a diode-array detector was used. As a stationary phase, an ACE 3 C18-AR column (150 mm × 4.6 mm, 3 μm particle size) protected by a guard column (Advanced Chromatography Technologies Ltd., Aberdeen, Scotland) was employed. An isocratic elution was applied using a mobile phase consisting of water/methanol 5:95 (*v*/*v*), with a constant flow rate of 1.2 mL/min and a column temperature kept at 35 °C. The injection volume was 20 μL and cholesterol was detected at a UV wavelength of 205 nm.

The bioaccessibility of cholesterol was calculated as follows:

Bioaccessibility (%) = (concentration of cholesterol in micellar phase/concentration of cholesterol in digestion medium) × 100

In order to determine the potential hypocholesterolemic effect, the bioaccessible cholesterol (total weight of cholesterol in micellar phase) was compared among the different samples (control, extracts and β-sitosterol, as a positive control). Any significant reduction in the bioaccessible cholesterol with respect to the control was considered a potential hypocholesterolemic effect. 

### 2.6. Evaluation of the Anti-Inflammatory Activity of Mealworm Extract in LPS-Stimulated Murine Microglial Cells

An immortalized microglia cell line of murine origin, obtained from A. Cuadrado, UAM, Spain, was used to test the anti-inflammatory activity of the mealworm extract. Microglial cells were maintained in the Roswell Park Memorial Institute (RPMI) 1640 medium supplemented with 10% heat-inactivated FBS, antibiotics (100 U/mL penicillin and 100 μg/mL streptomycin) and 2 mM L-glutamine at 37 °C in a humidified atmosphere with 5% CO_2_. Cells were pretreated for 2 h with 1% dimethyl sulfoxide (DMSO) as a vehicle or with the mealworm extract at the indicated doses (10, 25 and 50 µg/mL) in 2% FBS RPMI medium and further stimulated with bacterial lipopolysaccharide (LPS) (200 ng/mL) for 16 h. As controls, the cells received LPS or extract alone. Inducible nitric oxide synthase (iNOS) protein levels were analyzed by Western blot.

### 2.7. Evaluation of the Insulin-Sensitizing Activity of Mealworm Extract in Insulin-Stimulated Primary Mouse Hepatocytes

Primary mouse hepatocytes were isolated from the non-fasting C57Bl/6J male mice at 8–12 weeks of age by perfusion with collagenase as described [23]. Cells were seeded on 6- or 12-well collagen IV precoated plates (Corning Inc., Corning, NY, USA) and cultured in media containing Dulbecco’s modified Eagle medium (DMEM) and Ham’s F-12 medium (1:1) with heat-inactivated 10% FBS, supplemented with 2 mM glutamine, 15 mM glucose, 20 mM HEPES, 100 U/mL penicillin, 100 μg/mL streptomycin and 1 mM sodium pyruvate (attachment media)—maintained in this medium for 24 h. Then, the medium was changed to serum-free DMEM (5 mM glucose) with or without mealworm extract used at 25 and 50 µg/mL. The control cells received vehicle (1% DMSO). To analyze insulin signaling, after the incubation of the primary hepatocytes with the extract for 24 h, cells were stimulated with 5 or 1 nM insulin for 2, 5 and 10 min. The phosphorylation of the insulin receptor (IR) and protein kinase B (AKT) was determined by Western blot.

### 2.8. Western Blot Analysis 

To analyze iNOS protein levels and phosphorylation of IR and AKT (as indicated in 2.6 and 2.7), the cells were scraped off and incubated for 10 min on ice with lysis buffer (10 mM Tris-HCl pH 7.5, 5 mM EDTA, 50 mM NaCl, 30 μM sodium pyrophosphate, 50 mM sodium fluoride (NaF, S7920, Sigma-Aldrich, St. Louis, MO, USA), 100 µM o-vanadate sodium (S6508, Sigma-Aldrich), 1% Triton X-100, 1 mM phenylmethylsulfonyl fluoride (PMSF, P7626, Sigma-Aldrich) and 10 μg/mL protease inhibitors (P8340, Sigma-Aldrich), pH 7.4–7.6). Cellular lysates were clarified by centrifugation at 12.000× *g* for 10 min and after protein content was determined with the Bio-Rad Protein Assay Kit reagent (500-0006, Bio-Rad, Hercules, CA, USA). Protein samples (15–40 μg total protein) were boiled at 95 °C for 5 min in loading buffer (100 mM Tris pH 6.8, 10% glycerol, 4% sodium dodecyl sulphate (SDS), 0.2% bromophenol blue and 2 mM β-mercaptoethanol) and submitted to 8–10% SDS-PAGE. Gels were transferred to Immobilon membranes (Millipore, Burlington, MA, USA) and were blocked using 5% non-fat dried milk or 3% bovine serum albumin (BSA) in 0.05% Tween-20, 10 mM Tris-HCl and 150 mM NaCl pH 7.5, then incubated overnight with antibodies as indicated in 0.05% Tween-20, 10 mM Tris-HCl, and 150 mM NaCl pH 7.5. Immunoreactive bands were visualized with ECL chemiluminescent substrate (170-5061, Bio-Rad Laboratories, Hercules, CA, USA) developed in a ChemiDoc imager (733BR-3548, Bio-Rad Laboratories). The densitometric analysis of the bands was performed using ImageJ Software (NIH).

Primary antibodies used were: anti-phospho IR (Tyr1135/1136, ref. 3024), anti-total IR (ref. 3025), anti-phospho AKT (Ser473, ref. 4058), anti-total AKT (ref. 4691) purchased from Cell Signaling Technology (Danvers, MA, USA), anti-iNOS (ref. sc-650) and anti-vinculin (ref. sc-73614) purchased from Santa Cruz Biotechnology (Dallas, TX, USA) or anti-α-Tubulin (ref. T5168) purchased from Sigma Aldrich (St. Louis, MO, USA).

### 2.9. Evaluation of the Stimulatory Effect on Mitochondrial Respiration of the Mealworm Extract in Primary Bovine Aortic Endothelial Cells (BAECs)

Primary bovine aortic endothelial cells (BAECs) were used at passages 4–8. These cells were isolated from cow arteries obtained from an authorized slaughterhouse by treatment with collagenase A. Cells were cultured in glucose DMEM media (SIGMA) supplemented with 10% fetal bovine serum, 1% L-glutamine and penicillin/streptomycin at 37 ºC, 95% humidity and 5% CO_2_. Confluent BAEC cultures were treated with different mealworm extract concentrations (2.5 µg/mL, 5 µg/mL, 10 µg/mL, 20 µg/mL and 40 µg/mL) or an equivalent amount of dimethyl sulfoxide (DMSO) and vehicle (C).

Mitochondrial respiration was measured using a Seahorse XF24 analyzer (Agilent). A total of 90.000 BAECs were plated in Seahorse Bioscience V7 culture plates (Agilent) coated with fibronectin (10 µg/mL).

Extracellular oxygen flux was measured in DMEM medium (Sigma-Aldrich) with 5 mM D-glucose in the absence of pH regulators. Cells were incubated with this medium one hour before the beginning of the assay in an incubator at 37 °C and 0% CO_2_. Respiration was measured at baseline and after sequential treatment with 6 µM oligomycin, two successive doses of 150 nM FCCP and a mixture of 50 nM rotenone and 50 nM antimycin, taking three measurements per point. Between three and four technical replicates were included within each independent experiment.

### 2.10. Evaluation of the Stimulatory Effect on Mitochondrial Superoxide Production of the Mealworm Extract in Primary BAECs

A total of 350.000 BAECs grown to confluency (as indicated in 2.9) were seeded on fibronectin-coated cell imaging slides (EA DE 0030742060, Eppendorf, Hamburg, Germany,). Four hours after plating, the cells were treated with the extract as indicated in 2.9. Then, cells were incubated for 7.5 min with MitoSOX™ Red reagent (Thermo Fisher, Waltham, MA, USA), diluted in 1× HBSS with calcium chloride and magnesium chloride, at a final concentration of 3 µM. The MitoSOX™ Red reagent accumulates in the inner mitochondrial membrane and reacts with superoxide anion and fluoresces red with a maximum absorbance/emission of ~510/580nm. Cells were fixed with 4% paraformaldehyde and mounted with ProLong® Diamond (Thermo Fisher, Waltham, MA, USA). The images were taken using a Nikon i90 fluorescence microscope, DS-Qi1MC camera (Nikon Corp, Tokyo, Japan) using an mCherry filter and analyzed with Image J (NIH).

### 2.11. Antioxidant Activity of the Mealworm Extract by DPPH Assay

The antioxidant activity of the mealworm extract was studied by the DPPH radical scavenging assay according to Blois [24]. A total of 40 µL of the extract solution in methanol at 10 mg/mL was mixed with 560 µL of a DPPH solution in methanol (0.06 mM). The final concentration of the extract in the reaction medium was 0.6 mg/mL. Samples were incubated for 60 min in darkness at room temperature. The control samples were prepared in the absence of extract and following the same procedure. Absorbance was measured at 517 nm and methanol was used as a blank. Determinations were made in triplicate and the antioxidant activity was expressed as the percentage of DPPH inhibited according to the following formula:

% DPPH Inhibition = 100 − (Absorbance _sample_/Absorbance _control_) × 100

### 2.12. Statistical Analysis

Statistical analysis was performed using Microsoft Excel and Prism 8. Data normality was evaluated using the Kolmogorov–Smirnov test. Levene’s test was used for equality of variances. Statistically significant differences between the two groups was evaluated by two-tailed unpaired *t* test. Dose–response differences were also evaluated by one-way analysis of variance. Values were considered statistically significant at *p* < 0.05. Data in graphs are expressed as mean ± standard deviation (SD).

## 3. Results and Discussion

### 3.1. Chemical Characterization of the Mealworm Extract

The mealworm extract was analyzed by GC-MS after derivatization (formation of trimethylsilyl derivatives of all those less volatile compounds containing carboxyl or hydroxyl functional groups). This procedure allowed to tentatively identify 42 compounds, which were categorized into six major groups depending on their principal chemical family. Thus, lipids, nitrogen compounds, organic acids, carbohydrates, sterols and hydrocarbons were identified. The area percentage that each compound represents out of the total chromatographic area is also included in Table 1.

The highest area percentage corresponds to lipids (around 50%), whilst free fatty acids accounting for 23% of total chromatographic area. The most abundant free fatty acids were 9,12-octadecadienoic acid (linoleic acid) and (9Z)-octadecenoic acid (oleic acid), whereas among monoglycerides (26% total chromatographic area), 1-monopalmitin was the most abundant one. The free fatty acid profile of the extract agreed with the typical fatty acid profile of the fat of *T. molitor*. Concerning the interest in these fatty acids, the health properties of oleic acid are unquestionable. However, the effect of linoleic acid on human health still lacks consensus. Traditionally, it has been considered that proinflammatory mediators increase when the n-6/n-3 PUFA ratio is increased above 5 due to an excessive intake of n-6 PUFA, as is linoleic acid; in addition to other negative evidence, such as the risk of colonic inflammation [25]. However, a review recently re-visited the latest evidence for linoleic acid, and remarked the positive effects of increasing the dietary intake of linoleic acid on cardiovascular and cardiometabolic health [26].

Free amino acids were the second largest group of compounds (21% total chromatographic area) of the mealworm extract, proline being by far the most abundant one, with smaller amounts of tyrosine and pyroglutamic acid. Proline is an essential amino acid that seems to be an osmoprotective molecule that can modulate the intracellular redox environment and protect against oxidative stress [27,28]. Additionally, Andrade et al. [29] evaluated the effects of proline and LPS in the cerebral cortex and cerebellum on the activity of the expression of S100B and GFAP, oxidative stress parameters, enzymes of phosphoryl transfer network and mitochondrial respiration chain complexes. They found that the administration of proline did not alter the analyzed parameter in the cerebral cortex and cerebellum, in contrast to LPS administration. Additionally, the co-administration of proline and LPS showed the capacity of proline in preventing the effects of LPS. 

Other relevant compounds were identified in the extracts of potential interest, although presumably in low amounts due to the chromatographic abundance, include nonanedioic acid (or azelaic acid), a dicarboxylic acid widely used for dermatologic treatments and with interesting activities including its anti-inflammatory, antimicrobial and antioxidant potential [30,31]; as well as isocitric acid, which has gained attention in the pharmaceutical and food industry for its strong antioxidant activity [32]. The abundance of phosphoric acid and carbohydrates as disaccharides was also remarkable. 

The employed analytical procedure shows a partial characterization of a diversity of small to medium compounds containing –OH or –COOH functional groups; however, for a more complete and exhaustive characterization of the extract, other advanced analytical tools might be of interest. Despite this, the great diversity of chemical compounds of the mealworm extract is evidenced. Since the production of insect extracts is extremely scarce, the comparison of these results with previous studies is difficult. Nevertheless, these results were in agreement with a similar previous study focused on the ultrasound-assisted-extraction (UAE) of mealworm extracts [16]—the main difference being the different supplier of the mealworm larvae.

### 3.2. Inhibitory Activity against Pancreatic Lipase of Mealworm Extract

**Figure 1:**
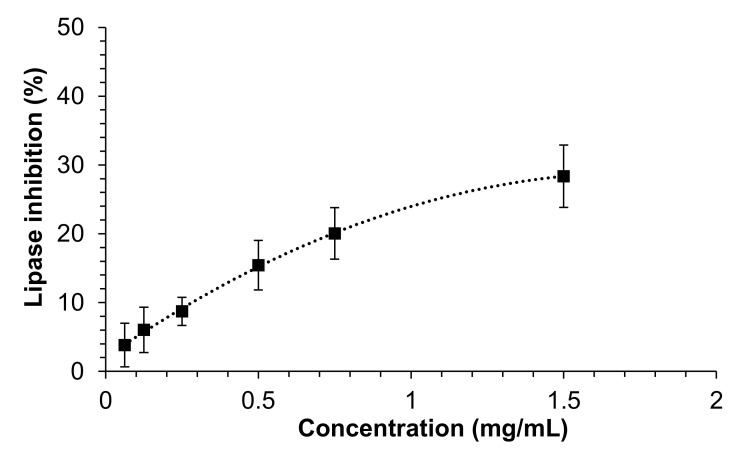
Effect of mealworm extract (mg/mL) on the inhibition (%) of pancreatic lipase enzyme under simulated intestinal conditions. Each data point corresponds to mean values ± SD, n = 3 independent experiments.

The ability of the extract to inhibit the pancreatic lipase in the small intestine is of key interest for blocking the absorption of lipids from the diet, and thus as a potential strategy against pathologies related to the metabolism of lipids, such as obesity, overweight, hypertriglyceridemia or hypercholesterolemia [33]. As shown in Figure 1, the extract was able to inhibit the activity of the pancreatic lipase in a moderate manner, although the concentrations assayed did not allow the estimation of the IC_50_ value. The maximum inhibition (28.4 ± 4.5%) was observed at 1.5 mg/mL, which was the highest studied concentration. In a previous work, we described for the first time the ability of insect extracts to inhibit the pancreatic lipase. In such a case, it was found that the IC_50_ of an extract from *T. molitor* obtained under the same conditions as those in the current study was approximately 0.7 mg/mL [16]. Such great difference in the inhibitory capacity between the two extracts might be explained by the fact that intestinal conditions were simulated during the assay in the present study, whereas in the previous work, no simulation of the physiological conditions was performed during the enzymatic assay. In this regard, we previously demonstrated that the addition to the reaction medium of bile salts, lecithin and other salts, in order to recreate a physiological environment, led to a notable decrease in the ability of diverse plant extracts to inhibit the enzyme [20]. These findings reveal once more the importance of determining the inhibitory activity of natural extracts against digestive enzymes under enzymatic conditions similar to those found in vivo as a way to evaluate, in a more realistic manner, the potential of natural extracts towards specific MetS-related prevention strategies. Another possible explanation for the lower inhibitory activity observed in the present study might be related to the different supplier (feeds and feeding regimes) of the insects between the two studies. 

### 3.3. Effect of Mealworm Extract on the Bioaccessibility of Cholesterol

**Figure 2:**
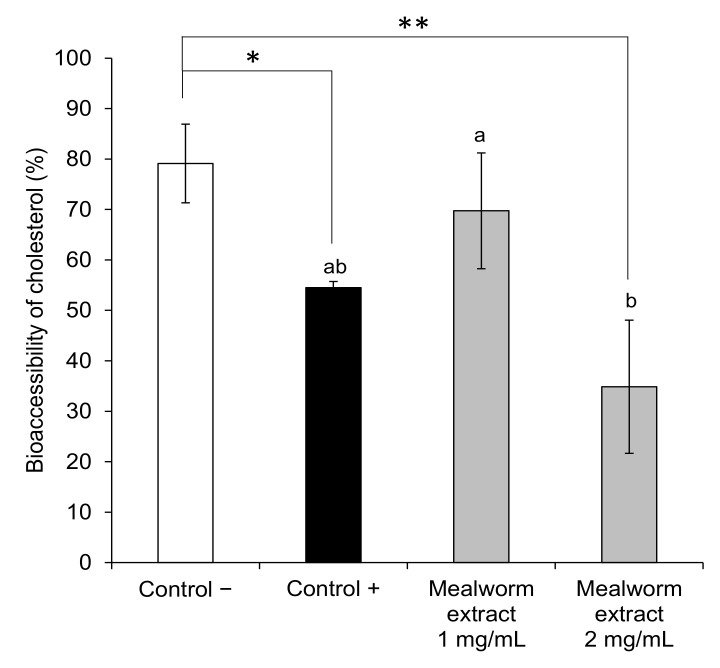
Bioaccessibility of cholesterol (%) after the gastrointestinal digestion of cholesterol in the absence (control –) and presence of inhibitors: β-sitosterol, as control + (at 1 mg/mL) and mealworm extract at 1 and 2 mg/mL. Bars are significantly different to control – when *p* = 0.014 (*) and *p* = 0.009 (**). Mean values with different letters (a−c) among inhibitors are significantly different if *p* ≤ 0.05.

The ability of the mealworm extract to interfere with the bioaccessibility of cholesterol and, consequently, its potential hypocholesterolemic effect was assayed by simulating in vitro the gastrointestinal digestion of cholesterol in the presence of the extract. Furthermore, a positive control (β-sitosterol) was used and a digestion without extract was performed as a negative control. The bioaccessibility of cholesterol at the end of the digestion in the presence of the mealworm extract at two different concentrations (1 and 2 mg/mL) is shown in Figure 2. First, the positive control expectedly reduced the bioaccessible cholesterol at the concentration assayed (1 mg/mL), which caused a 31% reduction (*p* = 0.014). This is because, in general, it has been assumed that plant sterols are able to reduce the intestinal absorption of dietary and biliary cholesterol by 30–50% [34]. Regarding the mealworm extract, it was able to significantly (*p* = 0.009) reduce the bioaccessibility of cholesterol by 55.9% at only the highest concentration assayed (2 mg/mL), and very interestingly, to an extent similar to that of the positive control. To the best of knowledge, this is the first study demonstrating the ability of a mealworm extract to interfere with the bioaccessibility of cholesterol after gastrointestinal digestion, suggesting a potential hypocholesterolemic effect by this mechanism. Others authors have recently shown the in vitro cholesterol-binding capacity of mealworm flour, as well as that of other edible insects [35]. In vivo, *T. molitor* has been demonstrated to decrease the serum total cholesterol in quails and mandarin fish fed with different levels of this insect, suggesting a link between the potential hypocholesterolemic activity and chitin contained in the mealworm [36,37,38]. Further studies elucidating the compounds contained in the mealworm extract responsible for the potential hypocholesterolemic activity might be of interest in order to develop insect food products aimed at enhancing this effect.

### 3.4. Effect of Mealworm Extract in Attenuating the Inflammatory Response Induced by LPS in Microglial Cells

**Figure 3:**
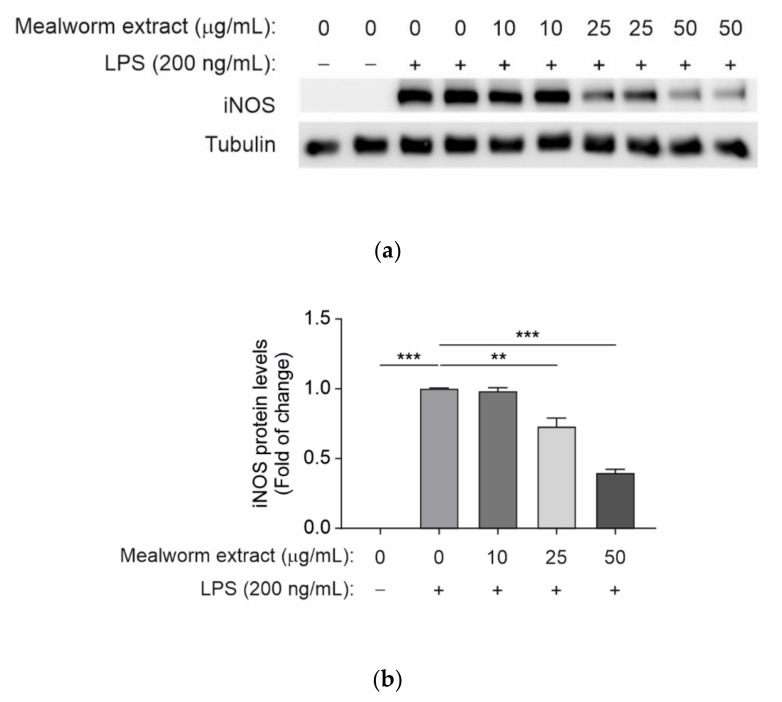
Mealworm extract treatment reduces proinflammatory iNOS protein levels in microglial cells upon LPS treatment. (**a**) An immortalized murine microglial cell line was pretreated with different doses (10, 25 and 50 µg/mL) of the mealworm extract for 2 h, before a 16 h stimulation with LPS (200 ng/mL) in 2% FBS RPMI medium. Representative Western blot images for iNOS are shown. Tubulin was used as a loading control. (**b)** Densitometric quantification of iNOS protein levels from A. For (b), values correspond to the mean ± SEM (*n* = 6). *p* < 0.01 (**), *p* < 0.001 (***) versus 0 µg/mL mealworm extract, according to one−way ANOVA followed by Tukey’s multiple comparison test.

Inflammation is a common cellular response in many diseases, as well as a defense response against pathogens. In particular, the chronic inflammation of the central nervous system (CNS) was related to peripheral metabolic alterations including those associated with obesity, insulin resistance and type 2 diabetes [39,40]. Therefore, the ability of the mealworm extract to counteract LPS-mediated proinflammatory effects in resident macrophages of the CNS was investigated in immortalized murine microglial cells. For this goal, cells were pretreated for 2 h with the mealworm extract at doses of 10, 25 and 50 µg/mL, after which LPS (200 ng/mL) was added to the culture dishes for a further 16 h. As controls, the cells were treated with LPS or the mealworm extract alone. At the end of the culture time, the pro-inflammatory response of the microglial cells was evaluated by analyzing the protein expression levels of iNOS. As shown in Figure 3, the mealworm extract was effective in decreasing the iNOS levels, as its anti-inflammatory effect is statistically significant at 25 and 50 µg/mL. These results strongly suggest that the mealworm insect extract might protect against neuroinflammation associated with obesity and type 2 diabetes. Evidence of non-protein extracts similar to that of the current assay has not been found, since most previous studies have focused on the major protein fraction. Thus, protein from mealworm larvae has been shown to markedly inhibit the production of NO without cytotoxicity in LPS-stimulated raw 264.7 macrophage cells and to reduce the expression level of cyclooxygenase-2 (COX-2) and iNOS protein through the regulation of mitogen-activated protein kinases (MAPKs) and nuclear factor kappa B [41]. Additionally, in LPS-induced RAW 264.7 cells, a mealworm water extract was demonstrated to decrease the production of NO in a dose-dependent manner, as well as reduce the expression of iNOS. Consequently, the protein levels of prostaglandin E2 (PGE2), iNOS, COX-2 and MARKs were significantly reduced [42].

### 3.5. Effects of Mealworm Extract on Mitochondrial Respiration and Superoxide Production

Changes in the inflammatory response may derive from a cell’s metabolic adaptations, particularly to the respiratory activity of mitochondria [43]. To determine whether the mealworm extract can affect the mitochondrial activity, we analyzed the effect of different doses of the extract on mitochondrial respiration and superoxide production, a by-product of mitochondrial oxidative activity.

**Figure 4:**
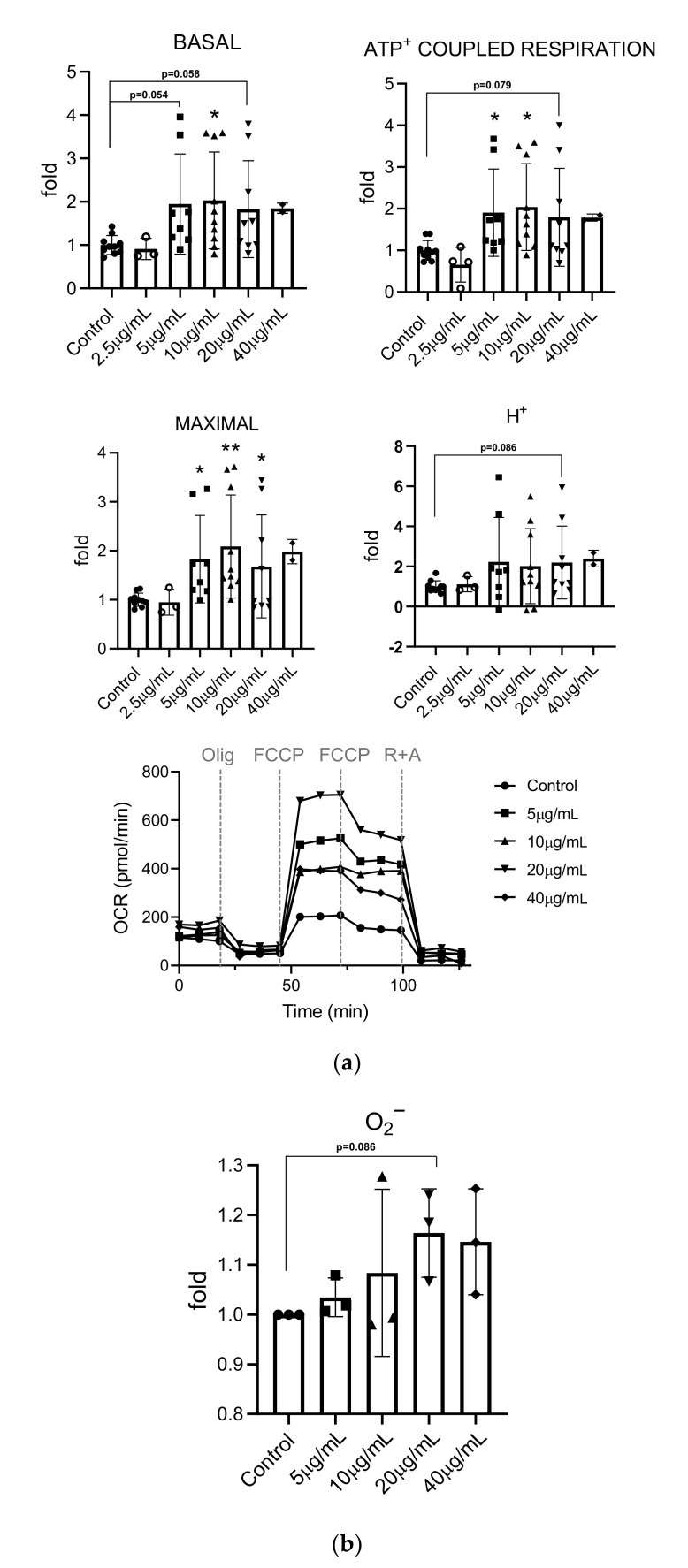
Effect of mealworm extract on mitochondrial respiration and mitochondrial superoxide production. (**a**) Confluent BAEC cultures were incubated with the indicated amounts of extract for 24 h. Mitochondrial respiration was determined using a Seahorse XF24 analyzer (Agilent). Oxygen consumption rates (OCRs) were used to determine basal respiration, maximal respiratory capacity, ATP^+^ coupled respiration, H^+^-leak (upper panels). A representative experiment showing the real-time changes in OCR of BAECs treated with the indicated amounts of extract for 24 h prior to the addition of oligomycin (Olig) (6 μM), FCCP (0.150 μM) and rotenone/antimycin A (R + A) (0.05 μM each) at the indicated times (bottom panel). The bottom panel shows a representative experiment; (**b**) confluent BAEC cultures were incubated with the indicated amounts of extract for 24 h and labelled with MitoSOX to determine mitochondrial superoxide (O_2_^−^) production. The graph shows mean values ± SD. n=3 independent experiments. Each data point is the average value of two technical replicates. *p* < 0.05 (*) *p* < 0.01 (**), versus Control, according to one-way ANOVA.

Basal oxygen consumption rates were measured under control conditions. The addition of oligomycin, an ATP synthase inhibitor, allowed to determine ATP-coupled respiration (ACR). The sequential addition of carbonilcyanide p-triflouromethoxyphenylhydrazone (FCCP), an electron transport chain (ETC) un-coupler, was used to determine the maximal respiration capacity. Finally, the injection of the rotenone (ETC Complex I inhibitor) and antimycin (ETC Complex III inhibitor) mixture allowed us to determine the non-mitochondrial respiration. The difference between maximal respiration and basal respiration indicated the coupling efficiency (CE) and the spare respiratory capacity. As shown in Figure 4, we observed an increase in basal (C vs. 5 µg/mL, *p* = 0.054; C vs. 10 µg/mL, *p* = 0.018; C vs. 20 µg/mL, *p* = 0.058), maximal (C vs. 5 µg/mL, *p* = 0.021; C vs. 10 µg/mL, *p* = 0.003; C vs. 20 µg/mL, *p* = 0.034) and ATP coupled respiration (C vs. 5 µg/mL, *p* = 0.045; C vs. 10 µg/mL, *p* = 0.011; C vs. 20 µg/mL, *p* = 0.079), which reached significance at 10 µg/mL. A non-significant increase in H+-leak levels (C vs. 20 µg/mL, *p* = 0.085) was also noted. 

We then analyzed the levels of mitochondrial superoxide (O_2_^−^) production (Figure 4). Under basal conditions, it is estimated that approximately 1–5% of the oxygen consumed by the cell is converted into reactive oxygen species (ROS) due to the escape of electrons from the ETC, which, when reacting with oxygen, produces a superoxide anion. Mitochondrial ROS production can increase under conditions of cellular stress. In response to extract treatment, we observed a non-significant increase in O_2_^−^ levels that could be related to the non-significant increase in H^+^-leak. This observation is also consistent with the higher observed OCRs rates.

### 3.6. Effect of the Mealworm Extract in the Early Steps of the Insulin Signaling Cascade in Primary Mouse Hepatocytes

**Figure 5:**
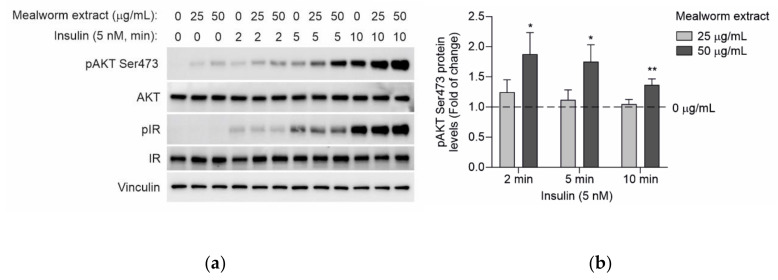

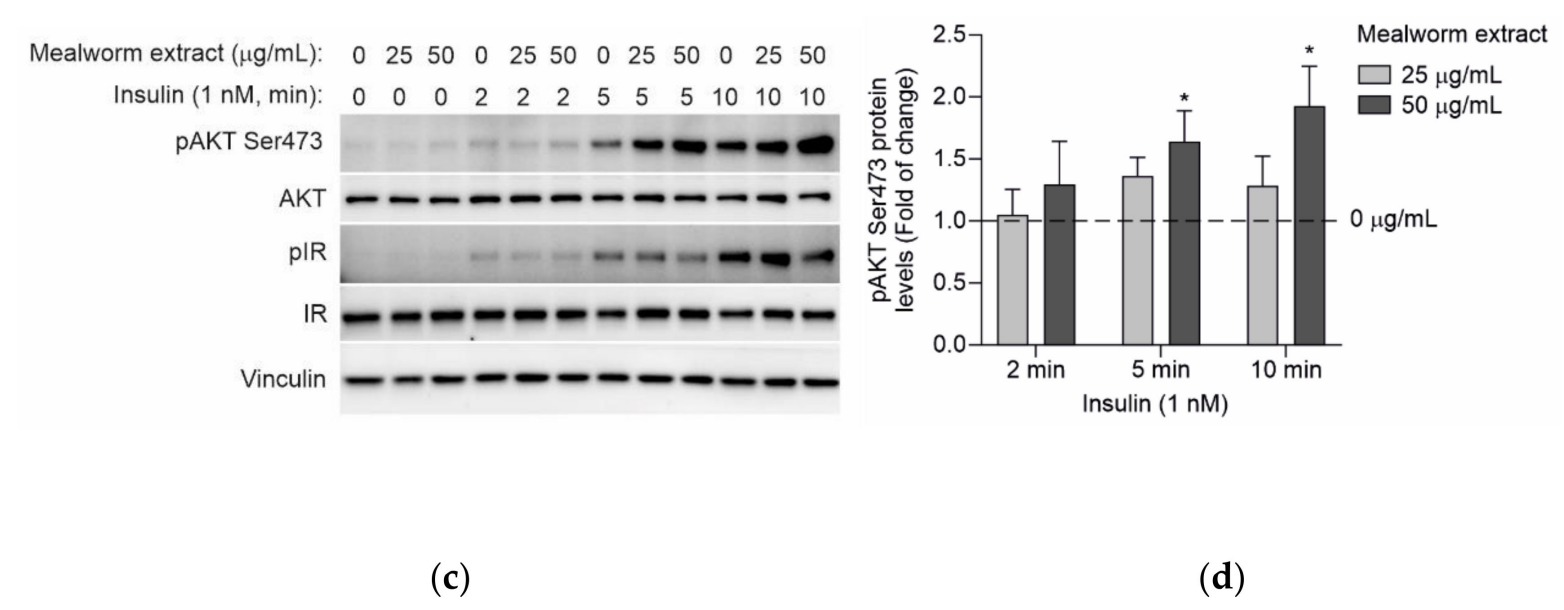
Mealworm extract treatment enhanced insulin signaling response in mouse primary hepatocytes. (**a**) Mouse primary hepatocytes were treated with mealworm extract at 25 and 50 µg/mL in serum−free DMEM (5.5 mM glucose) medium for 24 h prior to 5 nM insulin stimulation during the indicated time periods. Representative Western blot images for phospho-AKT (Ser473), total AKT, phopho-IR and total IR were shown. Vinculin was used as a loading control; (**b**) Densitometric quantification of phospho-AKT (Ser473) protein levels from A; (**c**) Mouse primary hepatocytes were treated with mealworm extract as described in A prior to 1 nM insulin stimulation. Representative images for phospho-AKT (Ser473), total AKT, phospho-IR and total IR were shown. Vinculin was used as a loading control; (**d**) Densitometric quantification of phospho-AKT (Ser473) protein levels from (**c**). For (**b**) and (**d**), values correspond to mean ± SEM (*n* = 6). *p* < 0.05 (*), *p* < 0.01 (**) versus 0 µg/mL mealworm extract, according to one−way ANOVA followed by Tukey’s multiple comparison test.

We investigated the effect of the mealworm extract on the insulin-mediated signaling cascade as a molecular readout to evaluate insulin sensitivity. To achieve this, we chose primary mouse hepatocytes due to their key role in insulin metabolic actions including the suppression of hepatic gluconeogenesis [44]. Primary mouse hepatocytes, cultured as described in the Materials and Methods, were pretreated for 24 h with the mealworm extract used at 25 and 50 µg/mL in serum-free DMEM medium (5.5 mM glucose). Then, the cells were further stimulated with 5 or 1 nM insulin for several time-periods (2, 5 and 10 min), after which the early steps of the insulin signaling cascade were analyzed. As shown in Figure 5, the insulin rapidly increased the phosphorylation of the insulin receptor (IR), as well as the phosphorylation of its downstream mediator AKT (also known as protein kinase B). Interestingly, the mealworm extract used at 50 µg/mL showed a statistically significant effect in enhancing insulin-mediated AKT phosphorylation in a time-dependent manner without affecting the phosphorylation of IR. The significant enhancement of insulin-mediated AKT phosphorylation by 50 µg/mL of the mealworm extract was observed when insulin was used at 5 nM as well as at 1 nM, a very low insulin concentration, evidencing the potent effect of the extract in enhancing the insulin sensitivity in hepatocytes. At the molecular level, these results highlight that the beneficial effects of the mealworm extract in potentiating AKT phosphorylation in primary mouse hepatocytes are elicited downstream of the IR. In agreement, this effect was also observed in diabetic C57BL/Ksj-db/db mice supplemented with an aqueous ethanol mealworm extract, similarly to that used in the current study, which significantly stimulated the phosphorylation of insulin receptor substrate-1 and AKT, as well as the activation of phosphatidylinositol 3-kinase in the insulin signaling pathway of skeletal muscles [45].

### 3.7. Antioxidant Activity of the Mealworm Extract

The potential antioxidant activity of the extract was evaluated by its ability to inhibit the DPPH· radical. The extract exhibited an inhibitory activity of 83.2 ± 0.7%, a value which was in agreement with the antioxidant activity of a UAE mealworm extract we previously produced under the same conditions (85% DPPH· radical inhibition) [16]. This is an interesting finding considering that the manufacturer of the raw insect material differed from one study to another, suggesting that the antioxidant activity of this specific insect species produced under the studied ultrasound conditions barely changes regardless of the rearing conditions.

## 4. Conclusions

The present study shows for the first time the in vitro potential of edible insects, and specifically, of a non-protein aqueous ethanol mealworm extract on the prevention and/or treatment of different MetS-related pathologies. The mealworm extract contains a variety of compounds, especially bioactive fatty acids such as oleic and linoleic acids, amino acids of importance such as proline and other compounds of potential interest such as azelaic acid and isocitric acid. In terms of its bioactivity, the extract has a strong antioxidant activity against the DPPH radical. It also exhibits a mild inhibition of the pancreatic lipase, a key enzyme responsible for the digestion of fats from the diets, as well as a very strong interference in the bioaccessibility of cholesterol, which is comparable to phytosterols as widely known hypocholesterolemic agents. Therefore, both mechanisms suggest the potential of this extract as a hypolipidemic product. Concerning its neuro anti-inflammatory potential, the extract decreases iNOS levels by counteracting LPS-mediated proinflammatory effects in murine microglial cells. It also increases basal, maximal and ATP-coupled respiration in primary bovine aortic endothelial cells, although the effect on mitochondrial superoxide production did not reach statistical significance, it may play a role in the induction of mitochondrial activity, and therefore, it deserves further investigation. Finally, a very strong effect of the mealworm extract is evidenced in terms of its ability to enhance insulin sensitivity via AKT phosphorylation in primary mouse hepatocytes. 

Therefore, the combined results of this study show that mealworm represents a novel source for producing value-added extracts with potential in the food and health industry as a candidate to treat MetS-related pathologies. However, a deeper understanding of the mechanisms underlying the effects described herein as well as the elucidation of the compounds responsible for the different bioactivities observed would be of high interest.

## Figures and Tables

**Table 1:** GC-MS characterization of mealworm (*Tenebrio molitor*) extract.

Rt (min)	Compound	Area	%
	LIPIDS		
	Fatty acids		
12.485	Dodecanoic acid	17,480	0.20
13.87	Tetradecanoic acid	78,440	0.88
15.02	Fatty acid n.i.	49,241	0.55
15.139	Hexadecanoic acid	438,621	4.93
16.143	9,12-Octadecadienoic acid	668,487	7.52
16.169	(9Z)-Octadecenoic acid	589,525	6.63
16.309	Octadecanoic acid	236,122	2.66
	Monoglycerides		
17.204	1-Monomyristin	41,992	0.47
18.029	2-Monopalmitin	143,652	1.62
18.202	1-Monopalmitin	1,113,769	12.53
18.958	2-Monostearin	106,180	1.19
19.131	Monoglyceride n.i. + sugar n.i.	941,077	10.59
			
	NITROGEN COMPOUNDS		
	Amino acids and derivatives		
8.908	Valine + amino acid n.i.	115,807	1.30
9.424	Leucine	55,652	0.63
9.625	Isoleucine	72,854	0.82
9.651	Proline	737,659	8.30
10.228	Serine	36,408	0.41
10.468	Threonine	30,762	0.35
10.719	Aspartic acid	12,740	0.14
11.55	Pyroglutamic acid	294,332	3.31
11.588	Amino acid n.i.	54,628	0.61
12.314	Amino acid n.i.	18,931	0.21
12.371	Amino acid n.i.	8630	0.10
14.569	Tyrosine	289,818	3.26
16.199	Tryptophan	145,443	1.64
	Non-protein nitrogen compounds		
9.112	Urea	33,290	0.37
15.637	Uric acid	35,977	0.40
			
	ACIDS		
9.461	Phosphoric acid	1,115,488	12.55
9.758	Butanedioic acid	21,627	0.24
13.42	Glycerophosphoric acid	83,584	0.94
13.537	Nonanedioic acid	59,739	0.67
13.811	Isocitric acid	158,515	1.78
			
	CARBOHYDRATES		
18.263	Disaccharide n.i.	297,470	3.35
18.483	Disaccharide n.i.	191,649	2.16
18.522	Disaccharide n.i.	180,864	2.03
18.672	Disaccharide n.i.	45,385	0.51
19.012	Sugar n.i.	92,244	1.04
19.209	Sugar n.i.	129,436	1.46
			
	STEROLS		
20.897	Cholesterol	108,886	1.22
			
	HYDROCARBONS		
17.692	Alcane n.i.	37,742	0.42

n.i. = not identified.

## Data Availability

The data presented in this study are available in this article or Supplementary Materials.

## Supplementary Materials

The following supporting information can be downloaded at: https://www.mdpi.com/2075-4450/13/10/896/s1, the whole blots of Western blot figures.
