# Supplementary material for: Effects of a Mealworm (Tenebrio molitor) Extract on Metabolic Syndrome-Related Pathologies: In Vitro Insulin Sensitivity, Inflammatory Response, Hypolipidemic Activity and Oxidative Stress
Source: Insects. 2022 Sep 30;13(10):896. doi: 10.3390/insects13100896 (PMC9604471; doi:10.3390/insects13100896)
Supplement: Supplementary file 1 [file insects-13-00896-s001.zip › insects-1848201-supplementary.pdf]

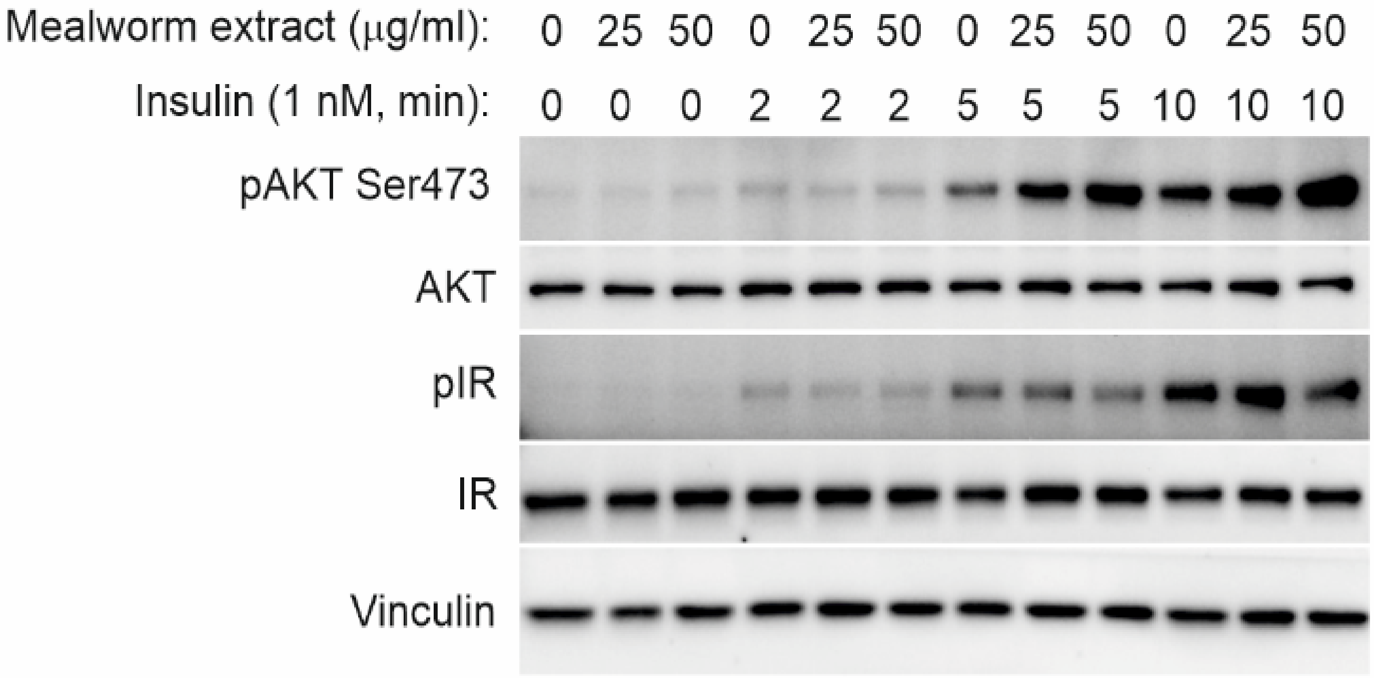

Mealworm extract (μg/ml): 0 25 50 0 25 50 0 25 50 0 25 50
Insulin (1 nM, min): 0 0 0 2 2 2 5 5 5 10 10 10
pAKT Ser473
AKT
pIR
IR
Vinculin

# Insulin 1nM

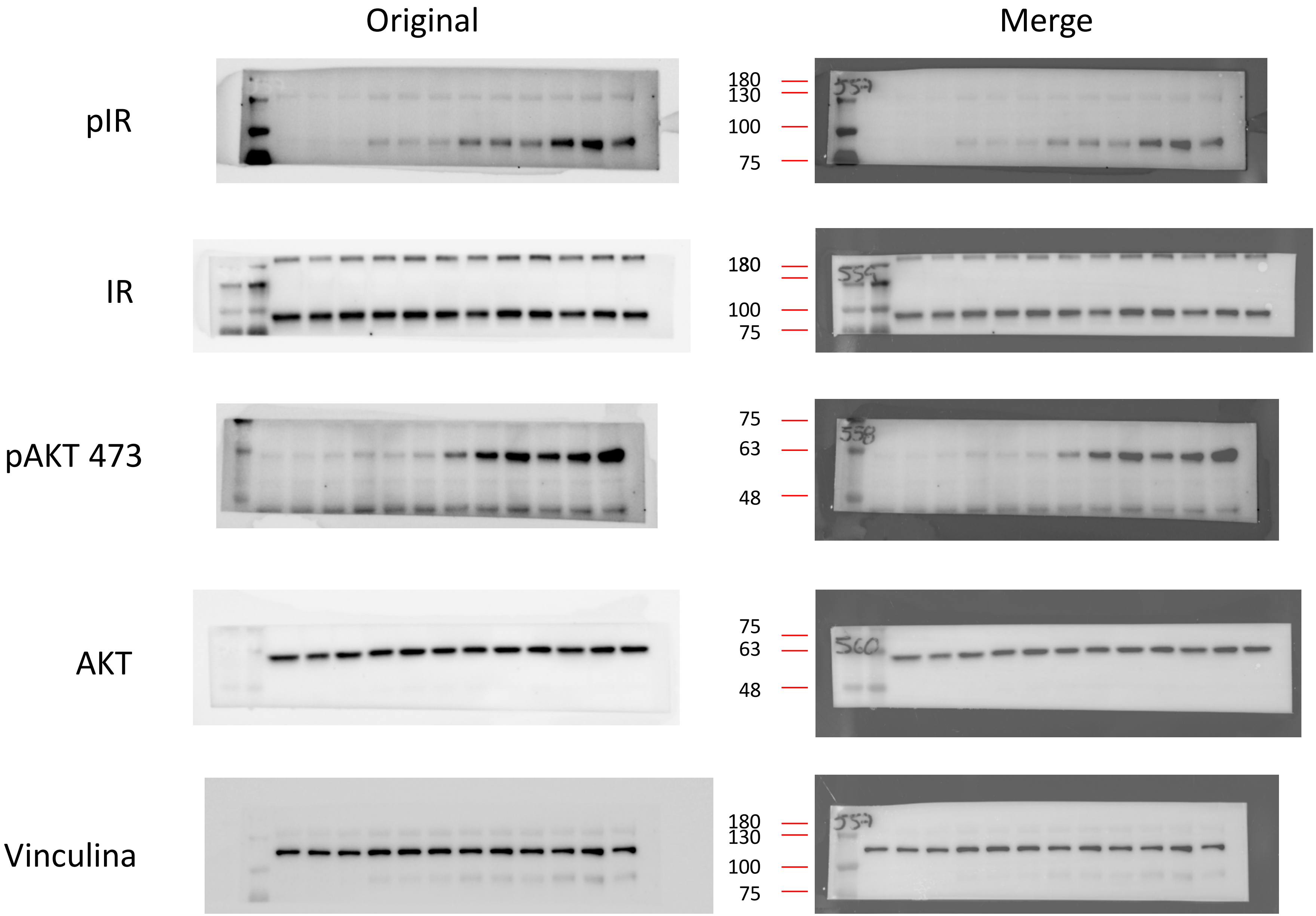
Original
Merge
pIR
180
130
100
75
IR
180
100
75
pAKT 473
75
63
48
AKT
75
63
48
Vinculina
180
130
100
75

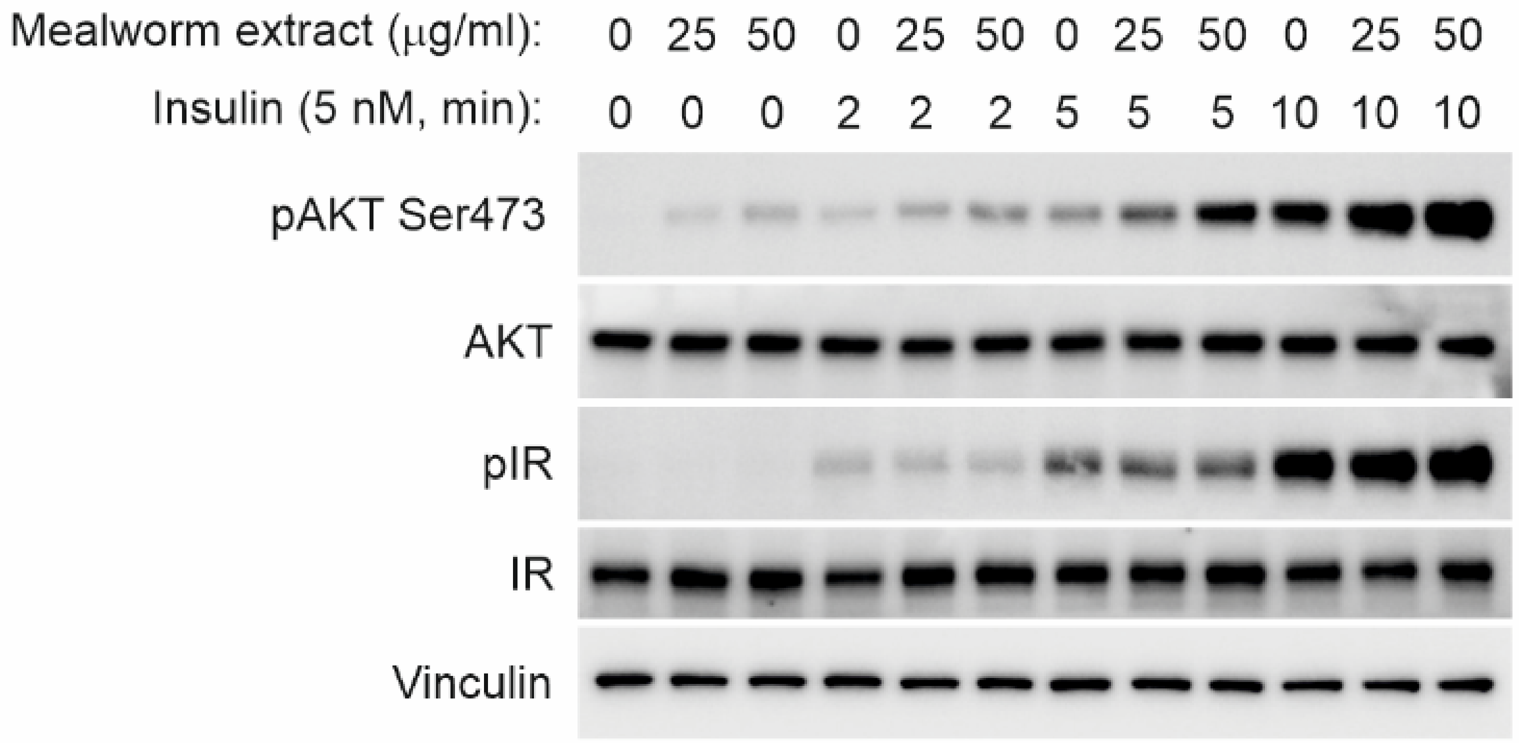
Mealworm extract (μg/ml): 0 25 50 0 25 50 0 25 50 0 25 50
Insulin (5 nM, min): 0 0 0 2 2 2 5 5 5 10 10 10
pAKT Ser473
AKT
pIR
IR
Vinculin

# Insulin 5nM

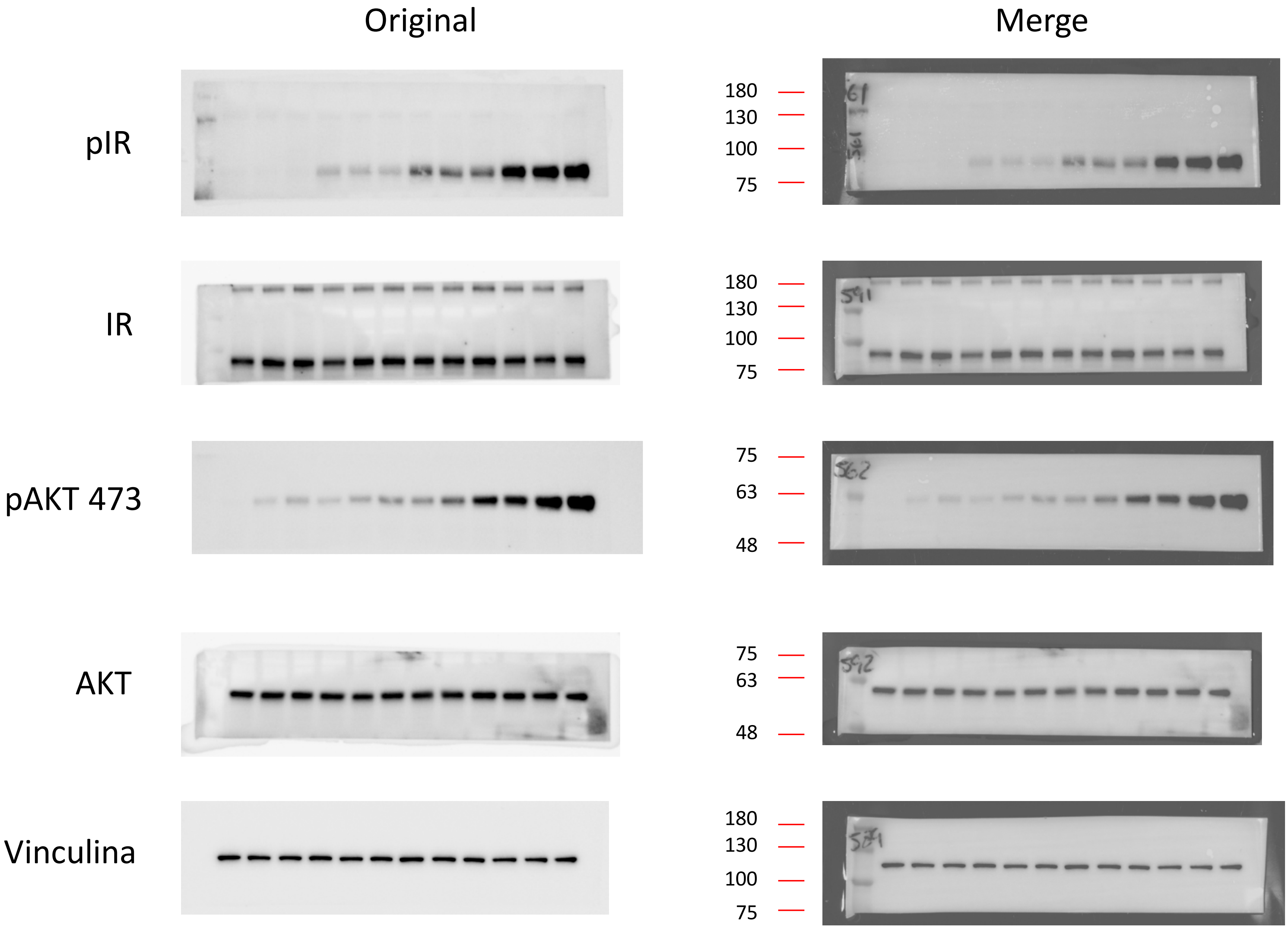
Original
Merge
pIR
IR
pAKT 473
AKT
Vinculina
180
130
100
75
180
130
100
75
75
63
48
75
63
48
180
130
100
75

**Microglia**

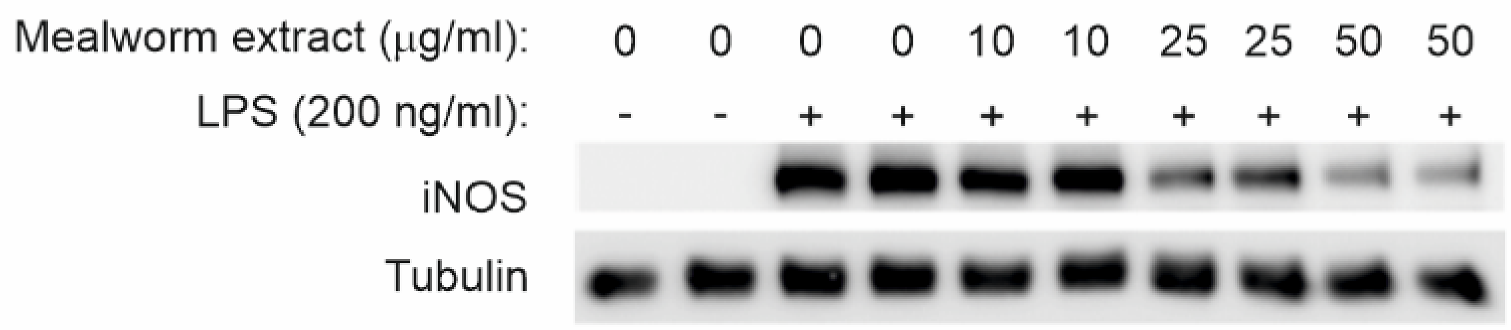
Mealworm extract (µg/ml): 0 0 0 0 10 10 25 25 50 50
LPS (200 ng/ml): - - + + + + + + + +
iNOS
Tubulin

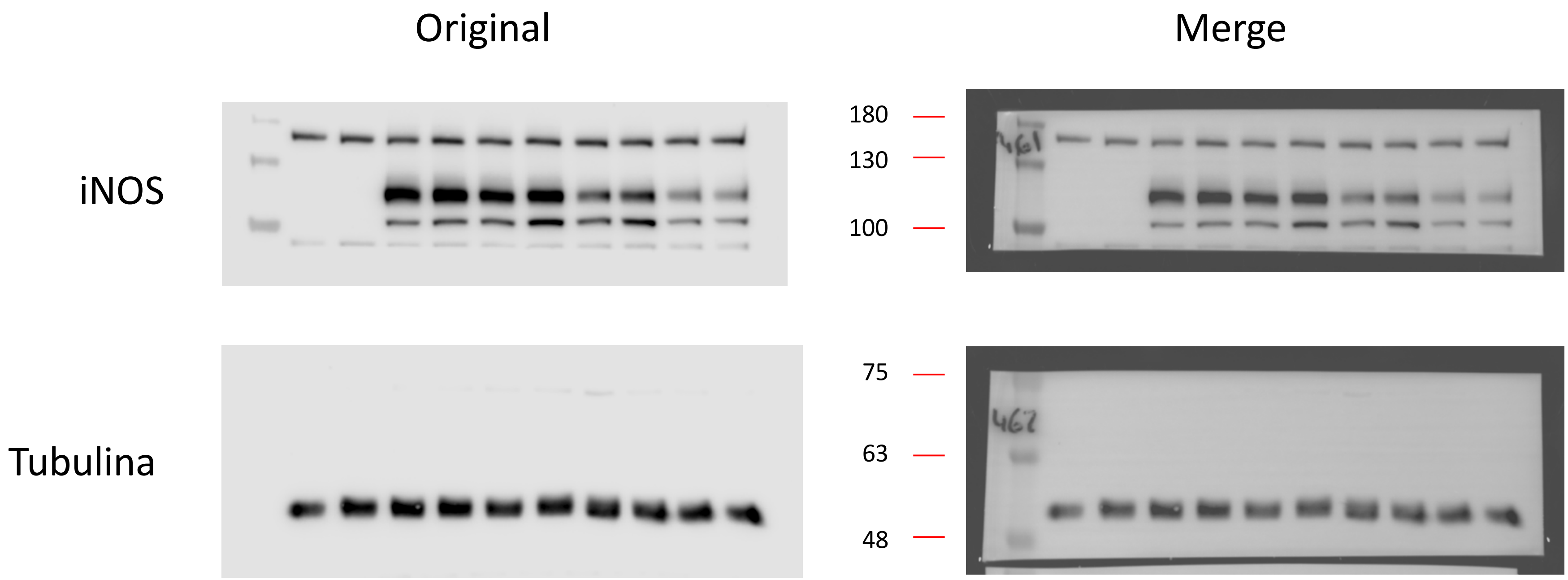
Original
Merge
iNOS
Tubulina
180
130
100
75
63
48
